# Reassessment of the prevalence of soil-transmitted helminth infections in Sri Lanka to enable a more focused control programme: a cross-sectional national school survey with spatial modelling

**DOI:** 10.1016/S2214-109X(19)30253-0

**Published:** 2019-07-19

**Authors:** Dileepa Senajith Ediriweera, Sharmini Gunawardena, Nipul Kithsiri Gunawardena, Devika Iddawela, Selvam Kannathasan, Arumugam Murugananthan, Channa Yahathugoda, Arunasalam Pathmeswaran, Peter John Diggle, Nilanthi de Silva

**Affiliations:** a Centre for Health Informatics, Biostatistics and Epidemiology, Faculty of Medicine, University of Kelaniya, Ragama, Sri Lanka; b Department of Public Health, Faculty of Medicine, University of Kelaniya, Ragama, Sri Lanka; c Department of Parasitology, Faculty of Medicine, University of Kelaniya, Ragama, Sri Lanka; d Department of Parasitology, Faculty of Medicine, University of Colombo, Colombo, Sri Lanka; e Department of Parasitology, Faculty of Medicine, University of Peradeniya, Peradeniya, Sri Lanka; f Department of Parasitology, Faculty of Medicine, University of Jaffna, Jaffna, Sri Lanka; g Department of Parasitology, Faculty of Medicine, University of Ruhuna, Galle, Sri Lanka; h Centre for Health Informatics, Computing and Statistics, University of Lancaster, Lancaster, UK

## Abstract

**Background:**

In Sri Lanka, deworming programmes for soil-transmitted helminth infections became an integral part of school health in the 1960s, whereas routine antenatal deworming with mebendazole started in the 1980s. A 2003 national soil-transmitted helminth survey done among schoolchildren found an overall prevalence of 6·9%. In our study, we aimed to reassess the national prevalence of soil-transmitted helminth infections to enable implementation of a more focused control programme that targets smaller administrative areas at risk of continued transmission.

**Methods:**

We did a cross-sectional, school-based, national survey using multistage stratified cluster sampling, covering all nine provinces as well as populations at high risk of soil-transmitted helminth infections living in urban slums and in plantation-sector communities. Our study population was children aged 5–7 years attending state schools. Faecal samples were collected and analysed with duplicate modified Kato-Katz smears. We modelled the risk of soil-transmitted helminth infection using generalised linear mixed-effects models, and we developed prevalence maps to enable informed decision making at the smallest health administrative level in the country.

**Findings:**

Between Jan 23 and May 9, 2017, we recruited 5946 children from 130 schools; 4276 (71·9%) children provided a faecal sample for examination. National prevalence of soil-transmitted helminth infection was 0·97% (95% CI 0·63–1·48) among primary schoolchildren. Prevalence in the high-risk communities surveyed was higher than national prevalence: 2·73% (0·75–6·87) in urban slum communities and 9·02% (4·29–18·0) in plantation sector communities. Our prevalence maps showed that the lowest-level health administrative regions could be categorised into low risk (prevalence <1%), high risk (prevalence >10%), or intermediate risk (1–10%) areas.

**Interpretation:**

Our survey findings indicate that the national prevalence of soil-transmitted helminth infection has continued to decline in Sri Lanka. On the basis of WHO guidelines, we recommend discontinuation of routine deworming in low-risk areas, continuation of annual deworming in high-risk areas, and deworming once every 2 years in intermediate-risk areas, for at least 4 years.

**Funding:**

Task Force for Global Health and WHO.

## Introduction

Mass deworming programmes directed against soil-transmitted helminths are among the largest public health programmes in low-income and lower-middle-income countries, as measured by coverage.^1^ WHO has reported that over 1 billion treatments that are effective against soil-transmitted helminth infections were delivered each year in 2015 and 2016.^2^, ^3^ Although existing WHO guidelines focus on morbidity control through mass deworming,^4^ modelling studies^5^ in the past decade suggest that it might be possible to interrupt transmission through expanded, community-wide, mass drug administration. As a result, global interest exists in whether a strategy targeting disease elimination might be feasible in some settings.^6^

The first island-wide survey^7^ of soil-transmitted helminth infection in Sri Lanka (then Ceylon), done in 1924–25, found that 90·5% of 32 507 people examined were positive for hookworm eggs. The following national survey^8^ of soil-transmitted helminth infections, done in 2003, involved the examination of 2713 schoolchildren from 144 schools. An overall cumulative prevalence of 6·9% was reported in that survey, with 4·0% prevalence of whipworm, 2·8% prevalence of roundworm, and 1·2% prevalence of hookworm. Much higher prevalences than those have been consistently reported in surveys done in the plantation sector of Sri Lanka: 89·7% in 1992,^9^ and 29·0% in 2009.^10^ Several previous studies^11^ have also shown that soil-transmitted helminth prevalences in urban slum dwellers are higher than those in rural areas of Sri Lanka. Both plantation sector and urban slums are known to have poor housing and sanitation compared with that of the rest of the country.

**Research in context**

**Evidence before this study**

Mass deworming programmes directed against soil-transmitted helminths are among the largest public health programmes in low-income and lower-middle-income countries, with treatment of 743 million people in 2017 and 638·5 million people in 2016, according to WHO estimates. We searched PubMed database for original studies of soil-transmitted helminths in Sri Lanka published with no date or language restrictions, using the search terms “soil-transmitted helminths” OR “soil transmitted helminths” OR “schoolchildren” AND “Sri Lanka”. Additionally, we searched for deworming programmes and control strategies between Oct 1, 2008, and Sep 30, 2018, using the search terms (“soil-transmitted helminths” OR “soil transmitted helminths”) AND (“deworming” OR “chemotherapy “ OR “mass drug administration” OR “elimination” OR “control”). Furthermore, we searched for risk mapping for soil-transmitted helminths prevalence between Oct 1, 2008, and Sep 30, 2018, using the search terms “soil-transmitted helminths” OR “soil transmitted helminths” AND “risk map” and manually searched the reference lists of articles identified. A national survey of soil-transmitted helminth infections in schoolchildren in Sri Lanka was last done in 2003, with an overall prevalence of 6·9% reported. This survey, together with a 2009 survey of schoolchildren in the plantation sector (a high-risk community), formed the basis for national deworming guidelines issued in 2012, where the nine provinces of Sri Lanka (the major administrative areas) were categorised as high or moderate risk. These guidelines recommended biannual treatment with single-dose mebendazole for all children in high-risk provinces (Uva, Sabaragamuwa, and Central Provinces) and annual treatment for children in moderate risk provinces (all other provinces). In our study, we re-assessed the prevalence of soil-transmitted helminth infections in Sri Lanka through a national survey of children attending state schools in 2017. We developed a statistical model based on data from the last national census of population and housing, to map the risk of soil-transmitted helminth infection at the smallest health administrative division level in Sri Lanka.

**Added value of this study**

National prevalence of soil-transmitted helminth infection has continued to decline in Sri Lanka, although prevalence remains higher than national values in the high-risk communities living in urban slums and the plantation sector. Prevalence mapping at the smallest health administrative division level suggested that these could be categorised as low risk (prevalence <1%), high risk (prevalence >10%), and intermediate risk (1–10%). Our results showed that a substantial number of health administrative divisions could be categorised as low risk, where routine mass deworming can be discontinued according to WHO guidelines.

**Implications of all the available evidence**

Deworming efforts in Sri Lanka could be targeted to selected regions that remain at high or intermediate risk of ongoing helminth transmission. Our national survey, mapping prevalence at the smallest health administrative division level in Sri Lanka, might facilitate the scaling down from a province-based national deworming programme to a more focused programme targeting smaller administrative areas at risk of continued transmission.

Key intervention strategies in Sri Lanka have included island-wide mass deworming programmes done in the 1930s,^7^ routine annual deworming of schoolchildren through the School Medical Inspection programme since the 1960s,^11^ routine deworming of pregnant women with mebendazole through state-run antenatal clinics since the 1980s,^12^ and mass biannual treatment of children in the plantation sector during 1994–2005.^10^ Therefore, Sri Lanka has had public health programmes to control these infections for nearly a century.^11^

In its latest effort, the Ministry of Health issued guidelines for deworming children and pregnant women in the community setting, for the 5-year period starting in 2013.^13^ These guidelines categorised the nine provinces (the major administrative areas) of the country as high or moderate risk, on the basis of the 2003 national survey^8^ of soil-transmitted helminth infections in schoolchildren together with the 2009 survey^10^ of schoolchildren in the plantation sector. These guidelines recommended biannual treatment with one dose of mebendazole for all children in high-risk provinces (Uva, Sabaragamuwa, and Central Provinces) and annual treatment for children in moderate-risk provinces (all other provinces).

In this study, we aimed to reassess the prevalence of soil-transmitted helminth infections in a national survey, done 4 years after implementation of these guidelines. We also developed a statistical model that would facilitate scaling down from a province-based national deworming programme to a more focused programme that targets smaller administrative areas at risk of continued transmission. This combined approach was adopted because a national survey that incorporated a sampling frame of the smaller administrative areas would have required unaffordable amounts of funding.

## Methods

### Study design and population

We did a cross-sectional, descriptive survey from Jan 23 to May 9, 2017. The survey covered all nine provinces and populations known to be at high-risk of soil-transmitted helminth infections living in low-income settlements in major cities and in the plantations in the central part of the country. The study population consisted of children attending Grade 1 and Grade 2 education in state schools (aged 5–7 years). Sri Lanka had 10 144 state schools in 2015, with a total population of 4·1 million students, including 1·73 million children in the primary cycle (Grades 1–5).^14^ More than 95% of school-going children in Sri Lanka attend state-run schools.^15^ All children attending the selected classes were considered eligible for inclusion in the study. There were no exclusion criteria.

Ethics approval was obtained from the Ethics Review Committee of the Faculty of Medicine, University of Kelaniya (P/124/09/2016). Written informed consent was obtained from parents or guardians of each child who participated in the study.

The cumulative prevalence of soil-transmitted helminth infections (ie, infection with one or more soil-transmitted helminths) was assumed to be less than 10% in all provinces. With simple random sampling, a sample size of 292 is required to obtain a 95% CI of ±2·5% for a prevalence estimate of 5%.^16^ Because we planned to use multistage stratified cluster sampling in this survey, this sample size was then multiplied by 1·5 (on the basis of an arbitrary design effect of 1·5), yielding a sample size of 438 per province. This was adjusted upwards to 500 to account for approximately 90% compliance, resulting in a total sample size of 4500 children in nine primary strata (provinces). A cluster was defined as one classroom (approximately 25 children), with 20 clusters per province. In addition to the primary strata, two supplementary strata of high-risk communities were added, each with a sample size of 500 children. The first supplementary stratum consisted of schools serving low-income settlements in urban areas, whereas the second stratum consisted of schools predominantly serving plantation-sector communities.

### Sampling frame

To select schools in the primary stratum, we used the national database of state schools maintained by the Ministry of Education. Schools were stratified within each of the nine provinces of Sri Lanka, according to the four functional types adopted by the Ministry. These types are defined by schools having classes for General Certificate of Education Advanced Level examination (type 1AB, which includes Science Stream, and type 1C), Ordinary Level examination (type 2), or Grade 1 to Grade 5 or Grade 8 only (type 3). The sample size for each province was distributed among schools on the basis of the relative size of the target student population in that particular stratum. This secondary stratification by school type was introduced to capture variation in the socioeconomic status of children attending these different types of school. From each substratum of types, schools were sampled by use of methods of probability proportional to size.

For the first supplementary stratum of schools, serving urban low-income settlements, we adopted purposive sampling to identify schools with the assistance of Medical Officers of Health attached to the respective Municipal Councils in the cities of Colombo (Western Province), Kandy (Central Province), Galle (Southern Province), and Jaffna (Northern Province), because the Ministry of Education database did not include this information. All such schools from different provinces were considered as one stratum. Because these schools were also included in the sampling frame of their respective provinces, data from this stratum were not used for calculating national rates.

The second supplementary stratum consisted of schools that predominantly serve plantation-sector communities. Plantation schools from Central, Southern, and Uva Provinces were identified by use of the list of schools in the database maintained by the Ministry of Education. Again, all such schools from different provinces were considered as one stratum. Because these schools were identified by use of information available in the national database of schools, they were removed from the sampling frame of their respective provinces and, therefore, the data from this stratum were used for calculating national rates.

We selected two clusters of children in each chosen school: one Year 1 class and one Year 2 class, selected randomly. All students in these classes were included. If the number of students in the selected classes was less than 25, the deficit was made up by adding another class from a neighbouring school of the same functional type. Therefore, the survey was designed to include approximately 5500 children in 220 clusters (180 from the primary strata and 40 from the two supplementary strata), attending at least 110 schools.

### Data collection and laboratory investigations

We collected samples between Jan 23 and May 9, 2017, before the start of the annual deworming and iron-folate supplementation programmes in schools. Data collection was done with use of both interviewer-administered questionnaires and laboratory investigations.

Selected students were provided with wide-mouthed, labelled containers for collection of faecal samples. Faecal examination for soil-transmitted helminth eggs was done within 6–8 h of collection in a hospital or medical faculty laboratory close to the selected school, using duplicate modified Kato-Katz smears (Kato-Katz kits; Vestergaard-Frandsen, Switzerland) with 41·6 mg faeces in each smear.^17^ Samples were left to clear for 20–60 min before reading. The mean egg count in each positive sample was multiplied by 24 to obtain a concentration of eggs per gram (epg) of faeces. Intensity of infection, calculated as epg of faeces, was categorised as light, moderate, or heavy intensity by use of cutoff values recommended by WHO.^18^

### Sociodemographic and environmental data

The geographical areas of the smallest health administrative level in Sri Lanka are the Medical Officer of Health (MOH) areas, which closely resemble the Divisional Secretariat Division (DSD) levels that are used for data collection during national censuses. Therefore, data aggregated at the DSD level were obtained from the Department of Census and Statistics, Sri Lanka for this part of the analysis. The following sociodemographic data were extracted from the National Census of Population and Housing done in 2012: population distribution, age distribution, education distribution, ethnic distribution, type of household, toilet facilities, type of toilets, and source of drinking water. We selected these variables because they are known risk factors for soil-transmitted helminth infection.^19^

Data on elevation and land cover were obtained from DIVA-GIS. Bioclimatic data (ie, annual rainfall, precipitation seasonality, annual mean temperature, maximum temperature in the warmest month, minimum temperature in the coldest month, and annual temperature range) were obtained from WorldClim. Normalised Difference Vegetation Index was obtained from the International Research Institute for Climate and Society (Columbia University, New York, USA). These data were attached to their respective DSD levels and used in the analysis as explanatory variables.

### Exploratory analysis

The stratified cluster sampling used was taken into consideration and we used appropriate sampling weights for calculating prevalence and 95% CIs at the provincial and national level.

The prevalence of any soil-transmitted helminths in schools was modelled with use of DSD-level data. The response variable was the number of students positive for one or more of the three soil-transmitted helminth infections among the total sampled at each school. All exposure variables were centred around the mean. We used generalised additive models to assess non-linear associations in explanatory variables. We then used generalised linear models to model the prevalence of soil-transmitted helminths at school level. Forward selection of explanatory variables was done with a likelihood ratio (deviance) test. We plotted standardised residuals against the fitted values to assess the lack of fit of the model. The fitted generalised linear model was then investigated for unexplained variability at school level by fitting a generalised linear mixed-effects model incorporating school-level random intercepts.

### Assessment of spatial correlation

We used school-level random effects to assess the residual spatial correlation with use of an empirical variogram.^20^ We calculated the variogram for the point predictions (conditional expectations) of the random effects and for 1000 random permutations of these. From the random permutations, we calculated pointwise 95% tolerance limits under the assumption of spatially uncorrelated random effects. The variogram of the point predictions was contained within the envelope of the tolerance limits throughout the plotted range of distances (appendix p 1), giving no evidence against the assumption of spatially uncorrelated random effects.

We used the fitted generalised linear mixed-effect model to predict soil-transmitted helminth prevalence, taking account of explanatory variables and school-level random effects. We developed a predictive grid surface to represent the landscape of Sri Lanka, as follows: the grid surface consisted of approximately 58 000 grid points at 0·01 decimal degrees spacing to cover the whole country; values of the explanatory variables were attached to each grid point; and 10 000 separate predictions were made at each of the grid points by first sampling from the multivariate normal distribution of the maximum likelihood parameter estimates and then sampling from corresponding predictive distribution of the random effects, to allow for the uncertainty in the parameter estimates.

Each predicted grid surface was overlaid with a polygon layer of MOH areas. All grid points pertaining to each MOH region were identified, and the predicted values at each grid point were used to calculate three summary measures of the spatial distribution of soil-transmitted helminth prevalence at MOH level. These summary measures—also called predictive targets—were the mean, upper quartile, and 90th centile of the spatial distribution of grid-point prevalence.

Probability contour maps at MOH level were then developed for each predictive target. A probability contour map shows the probability of the true value of a predictive target being less than (or greater than) any specified value. In our study, the probabilities of a predictive target being less than 1%, as well as greater than 10% were chosen for mapping. These cutoffs are in accordance with the decision tree set out in WHO's guide for managers of deworming programmes for children,^18^ which provides prevalence threshold-based guidance for decisions regarding the frequency of deworming when prevalence has been re-evaluated after several years of implementation of a deworming programme. Data analysis was done with R programming language, version 3.4.2. Technical details of the prediction algorithm are described in the appendix (p 6).

### Role of the funding source

The funder of the study had no role in study design, data collection, data analysis, data interpretation, or writing of the report. The corresponding author had full access to all the data in the study and had final responsibility for the decision to submit for publication.

## Results

Between Jan 23 and May 9, 2017, 5946 children were recruited from 130 schools. The number of schools surveyed was higher than planned because of inadequate numbers of students in some of the originally selected schools. 4276 (71·9%) of 5946 children provided a faecal sample (compliance rates by sampling stratum are provided in the appendix, p 2). The mean age of the study population was 6·1 years (SD 0·6). 3145 (52·9%) of children recruited were boys.

**Figure 1:**
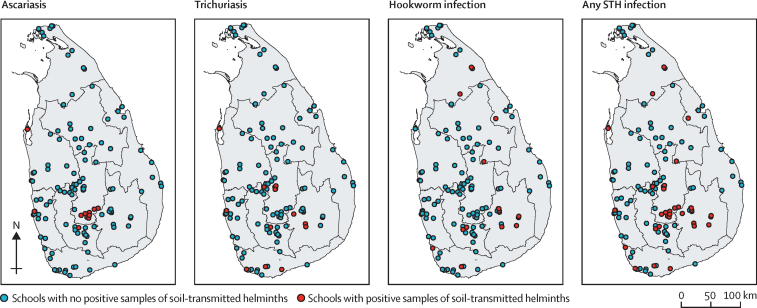
Geographical distribution of sampled schools and positive STH samples Demarcations within the country are the province boundaries. STH=soil-transmitted helminth.

Of the 4276 faecal samples examined for soil-transmitted helminth eggs, 77 samples from 33 schools were found positive (figure 1, table 1). The results of the soil-transmitted helminth survey indicate a national prevalence among schoolchildren in Sri Lanka of 0·97% (95% CI 0·63–1·48). However, this prevalence is higher among high-risk groups, with the highest prevalence in plantation sector communities, followed by Uva Province, urban slum communities, and the Southern Province. In all other provinces, prevalence was lower than 1·00% (table 1).

**Table 1:** Prevalence of STH infection by study stratum

		**Ascariasis**	**Trichuriasis**	**Hookworm infection**	**Any STH infection**
Provincial strata					
	Central	ND	0·42% (0·12–1·51)	ND	0·42% (0·12–1·51)
	Eastern	ND	ND	0·35% (0·05–2·32)	0·35% (0·05–2·32)
	Northern	ND	ND	0·61% (0·16–2·33)	0·61% (0·16–2·33)
	North Central	ND	ND	0·21% (0·03–1·53)	0·21% (0·03–1·53)
	North Western	0·22% (0·03–1·81)	0·22% (0·03–0·18)	ND	0·45% (0·06–3·57)
	Sabaragamuwa	ND	ND	ND	ND
	Southern	ND	0·67% (0·17–2·60)	0·45% (0·12–1·66)	1·12% (0·34–3·62)
	Uva	ND	1·48% (0·52–4·15)	1·48% (0·40–5·22)	2·96% (1·55–5·55)
	Western	ND	ND	ND	ND
High-risk strata					
	Plantations	8·04% (3·47–17·51)	0·20% (0·02–1·53)	1·18% (0·44–3·11)	9·02% (4·29–18·0)
	Urban slums	0·68% (0·02–3·76)	2·05% (0·43–5·89)	ND	2·73% (0·75–6·87)
Overall		0·45% (0·21–0·94)	0·25% (0·13–0·50)	0·29% (0·16–0·52)	0·97% (0·63–1·48)

Data are weighted percentages (95% CI). STH=soil-transmitted helminth. nd=not detected.

The majority of soil-transmitted helminth infections were due to *Ascaris lumbricoides* (43 of 77 infections detected), with most of these in the plantation sector (table 1). A third of 18 hookworm infections were found in children from the plantation sector, whereas the other infections were scattered among five provinces (table 1). Most of 16 *Trichuris trichiura* infections were found in Uva Province. Two mixed infections were found, both in a plantation sector school in Sabaragamuwa.

Of the 77 soil-transmitted helminth infections detected, the majority were of light intensity (table 2). 16 infections were of moderate intensity, and only one ascariasis infection was of severe intensity. Of the 17 children with infections of moderate to severe intensity, most (n=13) were from schools serving the plantation sector.

**Table 2:** Intensity of soil-transmitted helminth infections

	**Ascariasis (n=43)**	**Trichuriasis (n=16)**	**Hookworm infection (n=18)**
Light infection	29 (67%)	14 (87%)	17 (94%)
Moderate infection	13 (30%)	2 (12%)	1 (6%)
Heavy infection	1 (2%)	0	0

Data are number of infections (%).

Both fixed-effect and mixed-effect models showed that elevation, annual mean temperature, number of semi-permanent households, number of protective wells, and number of people living in estates in DSD divisions were significantly associated with soil-transmitted helminth prevalence. Individual effects of elevation and temperature did not show a significant association with soil-transmitted helminth prevalence, but their joint effects showed a significant association; therefore, both were included in the final model. The variance of the school-level random effect was significantly different from zero (Δ deviance 19·3, degree of freedom 1, p<0·0001).^21^ The plot of standardised residuals against fitted values did not suggest a lack of fit (appendix p 3).

When the explanatory variables were independently considered, increases in elevation, number of people living in the plantation sector, and number of semi-permanent households in DSD divisions showed positive associations with soil-transmitted helminth prevalence, whereas increases in annual mean temperature and number of protected wells within DSD divisions showed an inverse association (table 3). All variables, except the number of semi-permanent households, showed negative adjusted effects on soil-transmitted helminth prevalence in the mixed-effect model (table 3). Some pairs of explanatory variables were strongly correlated; therefore, the interpretation of adjusted effects needed to be done with care (appendix p 4). For example, the number of people living in the plantation sector showed a negative correlation with the number of protected wells and mean annual temperature. Therefore, a direct interpretation of the individual regression coefficients, which represent the effect of changing the values of one variable while the values of all others are held at fixed values, could be misleading.

**Table 3:** Parameter estimates of unadjusted effects and adjusted effects from the mixed-effect model

	**Estimate (SE)**	**Z value**	**p value**
**Individual effects**			
Elevation	0·00197 (4·61×10^−4^)	4·27	<0·0001
Estate population*	1·85×10^−5^ (4·68×10^−6^)	3·97	<0·0001
Semi-permanent households	8·78×10^−5^ (2·14×10^−5^)	4·11	<0·0001
Protected wells	–1·92×10^−4^ (6·86×10^−5^)	–2·80	0·0052
Annual mean temperature	–0·345 (0·0776)	–4·45	<0·0001
**Adjusted effects**			
Intercept	–5·54 (0·320)	–1·73	<0·001
Elevation	–0·0108 (0·00436)	–2·48	0·0129
Estate population*	–1·16×10^−4^ (2·95×10^−5^)	–3·94	<0·0001
Semi-permanent households	5·83×10^−4^ (1·38×10^−4^)	4·24	<0·0001
Protected wells	–2·13×10^−4^ (8·24×10^−5^)	–2·58	0·0098
Annual mean temperature	–1·83 (0·742)	–2·46	0·0139
Variance (random effects)	1·007	··	··

* Number of people living in estates.

**Figure 2:**
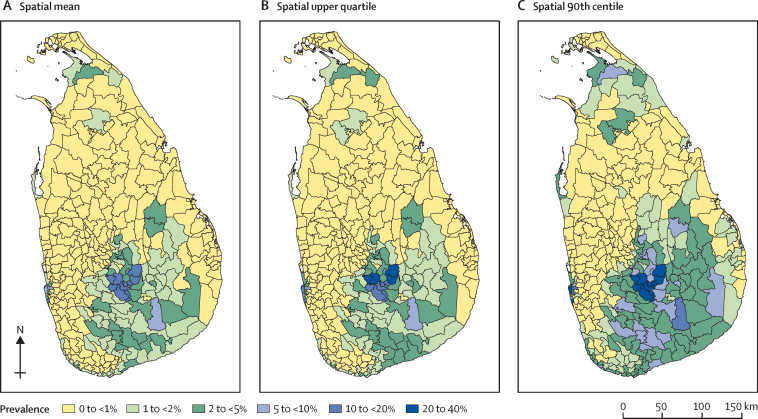
Estimated soil-transmitted helminth prevalence in MOH areas of Sri Lanka Prevalence is shown for each MOH area as spatial mean (representing the overall mean in the MOH area; A), spatial upper quartile (B), and spatial 90th centile (C). MOH=Medical Officer of Health.

**Figure 3:**
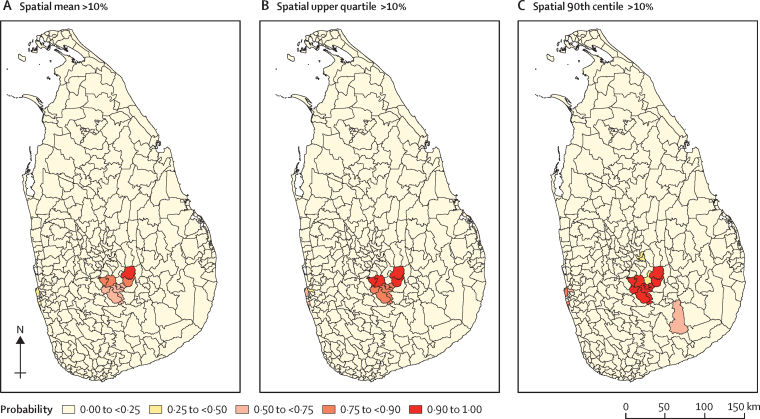
Probability of exceeding soil-transmitted helminth prevalence of 10% at Medical Officer of Health level Probability of exceeding soil-transmitted helminth prevalence of 10% for spatial mean (A), spatial upper quartile (B), and spatial 90th centile (C).

**Figure 4:**
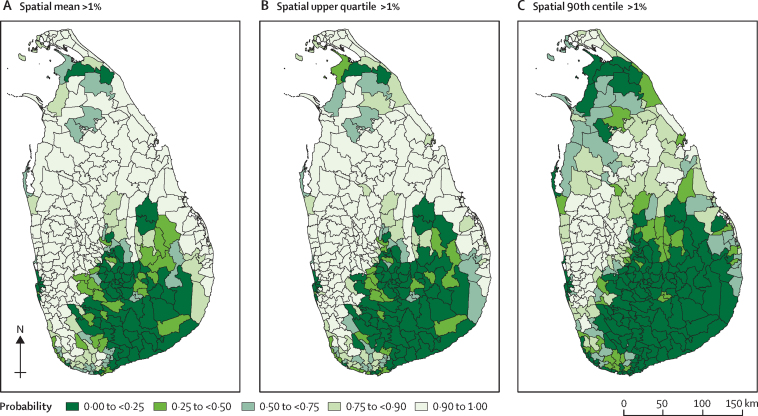
Probability of not exceeding soil-transmitted helminth prevalence of 1% at Medical Officer of Health level Probability of not exceeding soil-transmitted helminth prevalence of 1% for spatial mean (A), spatial upper quartile (B), and spatial 90th centile (C).

Maps for each estimated summary measure of soil-transmitted helminth infection prevalence in MOH areas are shown in figure 2. None of the MOH areas exceeded an estimated mean prevalence of 20% or higher (figure 2). Probabilities of exceeding 10% of prevalence at MOH areas (high risk) are shown in figure 3, whereas probabilities of not exceeding 1% of prevalence at MOH areas (low risk) are shown in figure 4. In 2017, 3·8 million school-going children were targeted for deworming. If the spatial mean of MOH areas is used for decision making purposes, as per WHO guidelines, only 103 000 children in high-risk areas would require continued annual deworming; another 1·5 million children in intermediate risk areas would require deworming once every 2 years; and routine deworming might be stopped for 2·1 million children living in low-risk areas.

## Discussion

In this study, we did a national survey of schoolchildren to estimate the prevalence of soil-transmitted helminth infections in Sri Lanka and map this prevalence at the smallest level of health administrative division. We also assessed prevalence in communities at high risk of soil-transmitted helminth infections. In the last national survey, done in 2003,^8^ the combined prevalence of helminth infections was 10% or higher in the Eastern, Northern, and Western Provinces and 1·0–9·9% in all other provinces. The survey of children attending plantation-sector schools, done in 2009,^10^ found a combined prevalence of 29·0%. The results of our survey suggest that soil-transmitted helminth prevalence has declined significantly from the previous levels that formed the basis of the deworming guidelines issued by the Ministry of Health in 2012.^13^ Moreover, most of the infections detected were of light intensity, and of the remaining infections, only one was of severe intensity. These figures suggest that even when soil-transmitted helminth infections do occur, they are unlikely to cause much morbidity.

Some limitations of our survey should be considered when interpreting these results. First, these prevalences are likely to be underestimates of the true prevalence, as studies^22^ in the past decade have shown that molecular techniques (eg, quantitative PCR [qPCR]) are more sensitive than microscopy-based techniques, such as the Kato-Katz smears, at low intensities of infection. Therefore, we used a modelling exercise as an alternative to an expensive laboratory-based survey.

Second, the sampling scheme was underpowered to detect prevalences lower than 1%. However, the 95% CI presented with each prevalence estimate and the probability contour maps presented provide a way of compensating for this limitation. The possibility of a selection bias arising from the purposive selection of schools serving urban low-income settlements and the compliance in providing faecal samples were considered in statistical modelling. In developing the statistical model, we adopted mixed-effect generalised linear models incorporating school-level random intercepts. These school-level random intercepts reflect the school-level specific characteristics that were not captured by measured covariates, including the differences in percentage of returned faecal samples (ie, compliance) and other natural and social environmental characteristics. These random effects add extra noise to and increase the uncertainty of predictions (ie, increasing SE), thereby avoiding spuriously precise inferences. Therefore, the adopted mixed-effect model accounted for possible biases and variability at cluster (ie, school) level. Although it was not possible to do a sensitivity analysis to compare the characteristics of schools and children in the final dataset with those of the potential sample population not included, a systematic bias seems unlikely because we found no significant correlation between compliance in providing faecal samples and soil-transmitted helminth prevalence (appendix p 2).

Soil-transmitted helminth infections are known to be focal in nature, and pockets of high prevalence might exist within larger areas of low prevalence. This pattern becomes accentuated when routine deworming programmes have been in place for some years, as is the case in Sri Lanka. Therefore, decisions regarding scaling down of a national programme should consider the existence of residual pockets of ongoing transmission in areas where housing and sanitation might be poorer than usual. The known high-risk communities in Sri Lanka were oversampled in our national survey through the introduction of additional sampling strata. However, the high cost of doing a field-based survey of this nature did not permit a sampling frame that would enable decision making at a level lower than the largest administrative divisions of the country. Therefore, we used the survey data to develop a statistical model that drew on possible risk factors for which data were already available from the last national census of population and housing. This approach provides policy makers and health administrators with information regarding the risk of soil-transmitted helminth infection at the lowest health administrative level.

WHO provides guidance for decisions regarding frequency of deworming for soil-transmitted helminth control, when prevalence has been reassessed after several years of implementation of a deworming programme.^18^ This guidance recommends that further preventive chemotherapy is not required when helminth prevalence is lower than 1%; that frequency of deworming might be reduced to once every 2 years in areas with prevalence between 1% and 10%; and that annual deworming should continue in areas with prevalence higher than 10%. Prevalence mapping at the level of health administrative divisions showed that a substantial number of MOH areas have soil-transmitted helminth prevalences lower than 1%. This suggests that the routine mass deworming strategy might be discontinued at national level and that deworming could be targeted to selected regions that remain at high risk of ongoing transmission.

Risk mapping of soil-transmitted helminth infections has been used in many countries to inform decisions regarding the launch of national or subnational deworming programmes.^23^, ^24^, ^25^ However, very little literature exists on scaling down these programmes. We developed three types of prevalence maps at MOH level to facilitate informed decision making. The mean prevalence map helps to assess the overall status, whereas upper quartile and 90th centile maps are useful to identify residual pockets within MOH areas. The precision of prevalence estimates can vary in different MOH areas and might cause uncertainty in decision making. Therefore, we developed separate probability maps to facilitate more evidence-based decision making. These probability maps quantify the likelihood of exceeding 10% and not exceeding 1% prevalence of soil-transmitted helminth infections at the MOH areas and aid the identification of high-risk and low-risk regions with confidence. For example, if very high precision is required for decision making, then estimates with very high probability (ie, ≥0·9) can be used. If low precision is acceptable for decision making, then a minimum probability of 0·75 can be used.

The three different kinds of maps developed can be used depending on the decision at hand. For example, mean prevalence maps could be used if the decision to stop or continue routine deworming is to be based on overall average prevalence of infection in the target MOH area. Similarly, upper quartile and 90th centile maps could be used if the decision making needs to address any residual soil-transmitted helminth pockets within the MOH areas. These calculations show how the choice of a summary measure can have substantial effect on MOH-level inferences, underlying the importance of thinking carefully about the relevant predictive target for decision making.

The scaling down or discontinuation of routine deworming programmes could lead to a rebound in infection if the transmission has not been reduced to a level at which helminth reproduction can no longer occur and the helminth population collapses without further treatment of remaining infections.^5^ The WHO-endorsed strategy for soil-transmitted helminth control focuses on initial elimination of infections of moderate to severe intensity to control morbidity and recommends cessation of routine deworming when prevalence of soil-transmitted helminth is lower than 1%, as measured by the Kato-Katz technique.^18^ However, the past decade has seen a shift in global interest towards achieving elimination of transmission instead of morbidity control alone.^5^, ^6^, ^26^ Modelling studies^5^, ^27^ have suggested the possibility of interrupting transmission of soil-transmitted helminths through intensive community-wide chemotherapy when high coverage (>80%) and compliance are achieved. The DeWorm3 Project^28^ set out to test the hypothesis that transmission can be interrupted if repeated annual mass drug administration covering all age groups is successful in reducing prevalence (as measured by qPCR) to less than 2%.^28^ However, the type of sampling and molecular diagnostics used in DeWorm3 are not affordable options for policy making decisions in most developing countries, even in those that have reached the level of socioeconomic development that usually accompanies declining prevalence of soil-transmitted helminth infections.

Rapid reinfection with soil-transmitted helminths, and the predisposition of some individuals to repeated infections, limits the power of deworming to a short-term strategy for the control of soil-transmitted helminth infections. Long-term solutions require improvements in water and sanitation sectors. In South Korea, massive economic development together with accompanying improvements in sanitation and hygiene probably helped to further decrease soil-transmitted helminth transmission.^29^ In Sri Lanka as well, substantial improvements in living conditions and socioeconomic status have occurred in most areas of the country, including in urban slums and plantations. Only 3·0% of plantation, 1·3% of rural, and 0·4% of urban households reported not having access to sanitary facilities.^30^

Debates have occurred in Sri Lanka among paediatricians, physicians, community health physicians, medical parasitologists, health administrators, and policy makers regarding the wisdom of adopting a strategy that pursues the complete elimination of soil-transmitted helminth infections. Emerging evidence^31^, ^32^ has shown that infections with soil-transmitted helminths, which have been a part of the human gut microbiota for millennia, might confer some health benefits that arise from immune modulation. Health policy makers were also reluctant to agree to the funding allocations that would be necessary for the implementation of such an elimination strategy when other national health needs are present, including a sharp increase in non-communicable diseases and an ageing population.

In this context, a targeted approach seems the most prudent way forward for Sri Lanka. Strategies can vary according to risk of infection, but deworming should be continued until the risk of rebound is minimal. Our findings suggest that the MOH areas might be categorised into low risk (prevalence <1%) areas where routine deworming might be stopped, high risk (>10%) areas where annual deworming would continue, and intermediate risk (1–10%) areas where children would require deworming once every 2 years. At the end of a period of 4 years, a repeat survey might be done, and data combined with risk mapping on the basis of the next census of population and housing, to facilitate evidence-based decision making.
