# Supplementary material for: Reassessment of the prevalence of soil-transmitted helminth infections in Sri Lanka to enable a more focused control programme: a cross-sectional national school survey with spatial modelling
Source: Lancet Glob Health. 2019 Jul 19;7(9):e1237–46. doi: 10.1016/S2214-109X(19)30253-0 (PMC6688098; doi:10.1016/S2214-109X(19)30253-0)
Supplement: Supplementary appendix [file mmc1.pdf]

THE LANCET
Global Health

Supplementary appendix

This appendix formed part of the original submission and has been peer reviewed. We post it as supplied by the authors.

Supplement to: Ediriweera DS, Gunawardena S, Gunawardena NK, et al. Reassessment of the prevalence of soil-transmitted helminth infections in Sri Lanka to enable a more focused control programme: a cross-sectional national school survey with spatial modelling. *Lancet Glob Health* 2019; published online July 19. http://dx.doi.org/10.1016/S2214-109X(19)30253-0.

Supplementary figure 1

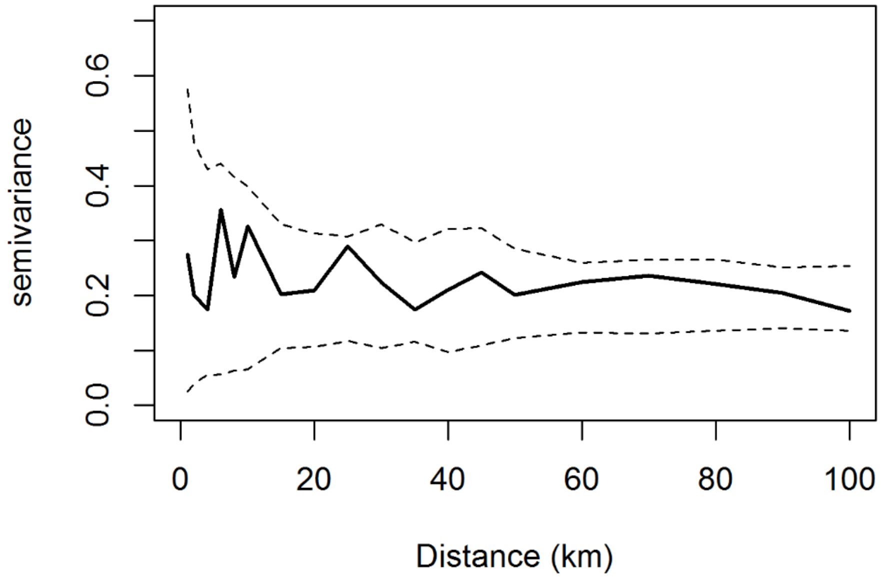

Supplementary figure 1: Empirical variograms of school level random effects. The black line indicates the empirical variogram of the predicted random effects. Dashed lines indicate the 95% pointwise tolerance envelope for the empirical variograms of 1000 random permutations of the predicted random effects.

Supplementary table 1: Number of schools, students and faecal samples in each stratum

	Number of schools included in survey	Number of students recruited to study	Number of faecal samples received	Compliance rate in returning faecal samples (%)	Estimated prevalence of STH infection (95% CI)
Central Province	13	617	478	77.5	0·42 (0·12-1·51)
Eastern Province	10	429	288	67.1	0·35 (0·05-2·32)
Northern Province	11	500	329	65.8	0·61 (0·16-2·33)
North Central Province	11	643	468	72.8	0·21 (0·03-1·53)
North Western Province	12	587	445	75.8	0·45 (0·06-3·57)
Sabaragamuwa Province	14	576	393	68.2	n.d.
Southern Province	11	596	446	74.8	1·12 (0·34-3·62)
Uva Province	11	585	406	69.4	2·96 (1·55-5·55)
Western Province	10	540	367	68.0	n.d.
Plantations	14	624	510	81.7	9·02 (4·29-18·0)
Urban slums	11	249	146	58.6	2·73 (0·75-6·87)
Total	128	5,946	4,276	71.9	0·97 (0·63-1·48)

Supplementary figure 2

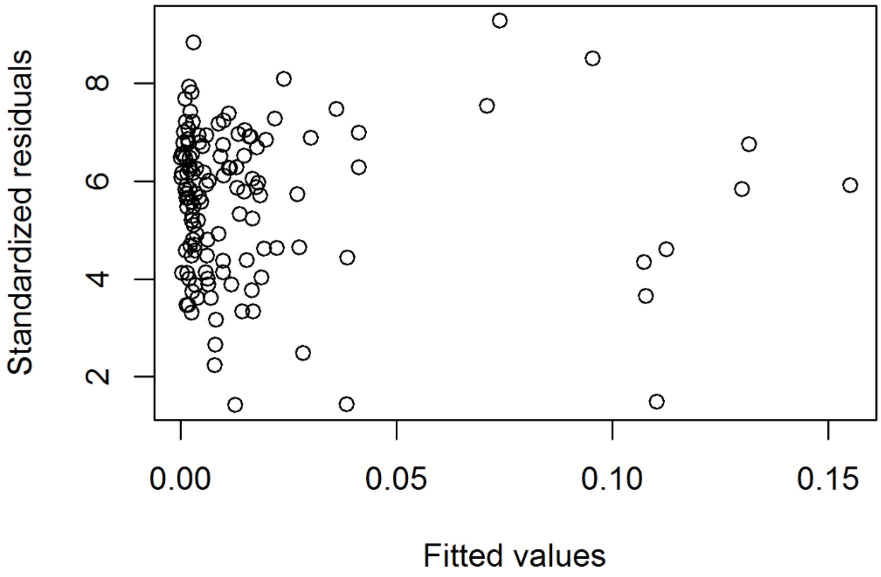

Supplementary figure 2: Scatter plot of school level standardized residuals against fitted values of the fitted fixed effect model.

Supplementary figure 3

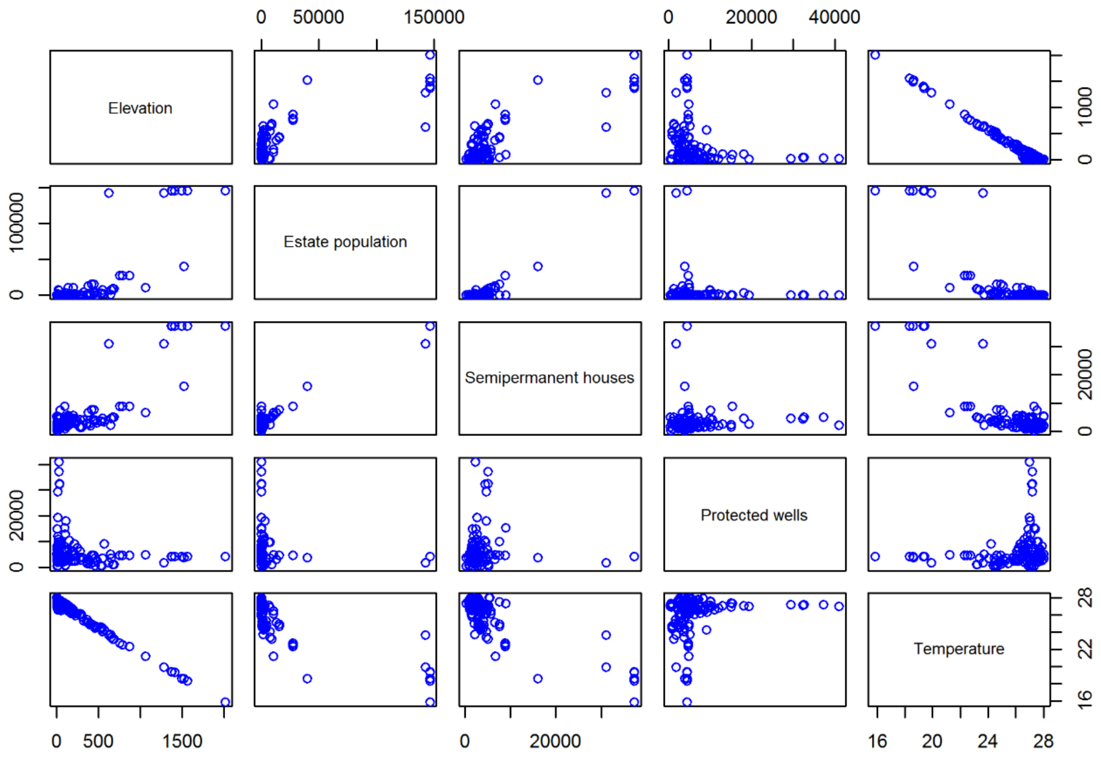

Supplementary figure 3: Scatterplot between explanatory variables. Note that there are inverse relationships between explanatory variables (e.g. elevation and estate population versus temperature and protected wells) and positive relationships between variables (e.g. elevation, estate population and semi-permanent houses).

Predictive inference and the prediction algorithm

A.1 Predictive inference

Let P(x) denote the spatially continuous prevalence surface over Sri Lanka

The *spatial mean* prevalence over a region A with area |A| is

$$\overline{\mathrm{P}} = |A|^{-1} \int_A P(x)\, dx$$

The *spatial upper quartile* is the value *P75* such that *P(x)* is less than *P75* over 75% of A. Similarly, the spatial 90th centile, P90, is the value such that P(x) is less than P90 over 90% of A.

The true prevalence surface, and hence $\overline{\mathrm{P}}$, P75 and P90, are unknown. We call $\overline{\mathrm{P}}$, P75 and P90 our *predictive targets*. Our incomplete knowledge about a predictive target is represented by its *predictive distribution*, i.e. the probability distribution of the target conditional on the data that we used to fir our model. The mean of the predictive distribution is conventionally used as a point prediction of a target, i.e. a single “best guess". This choice makes the mean square prediction error as small as possible. Other useful summaries are the predictive probabilities that the target does or does not exceed a specified value.

More often than not, the predictive distribution of a target, *T* say, is mathematically intractable. In such cases, we simulate a large number of values $T_1, \ldots, T_n$ from the predictive distribution and use this sample to approximate the required summaries.

A.2 Predictive algorithm

In the current context, we approximate the continuous surface *P(x)* over the whole of Sri Lanka by a grid of points at a spacing of 0.01 decimal degrees, and proceed as follows.

1. First draw a sample of parameter values from the multivariate Normal distribution of the maximum likelihood estimators for all parameters of the fitted multiple mixed-effect model (i.e. table 3 – adjusted effects).
2. For each set of sampled parameter values, draw a sample of values for the random effects, and hence for $P(x_{ij})$, from their predictive distributions, where x_{ij} is the jth grid-points that fall within the ith MOH.
3. For each MOH calculate the corresponding values of the predictive targets $\overline{P}$, *P75* and *P90* as the sample means, upper quartiles and 90th centiles of the values of $P(x_{ij})$ at grid-points x_{ij} that fall within the ith MOH.
4. Repeat steps 1 to 3 ten thousand times. Call the resulting values of the three targets $\overline{P}_{ik}$, $P75_{ik}$ and $P90_{ik}$ where *i* denotes an MOH and *k* = 1, 2,….,,10000.
5. Use the values $\overline{P}_{ik}$ to construct predictive maps as follows.
   (a) The point predictive map shows, for the ith MOH, the sample mean,
   $\overline{P}_i = (\sum_{k=1}^{10000} \overline{P}_{ik})/10000$.
   (b) The probability contour map (PCM) for non-exceedance of 1% prevalence shows, for the ith MOH, the proportion of the $\overline{P}_{ik}$ that are less than 0.01.
   (c) The PCM for exceedance of 10% prevalence shows, for the ith MOH, the proportion of the $\overline{P}_{ik}$ that are greater than 0.10.
6. Repeat step 5 using the values of $P75_{ik}$ in place of $\overline{P}_{ik}$.
7. Repeat step 5 using the values of $P90_{ik}$ in place of $\overline{P}_{ik}$.
